# Assessing Motor Function in Frail Older Adults in Their Home Settings: Challenges, Strategies and Recommendations

**DOI:** 10.3390/ijerph20156515

**Published:** 2023-08-03

**Authors:** Lijuan Yin, Maria Caceres, Jordan Skowronski, Naoko Muramatsu

**Affiliations:** 1 Institute for Health Research and Policy, University of Illinois Chicago, Chicago, IL 60608, USA; cacemari@uic.edu (M.C.); jskowr3@uic.edu (J.S.); 2 School of Public Health, University of Illinois Chicago, Chicago, IL 60612, USA

**Keywords:** functional limitations, physical activity, home care, in-home assessment, intervention outcomes, home environment, safety, interviewer observation, data management

## Abstract

Assessing motor competence is essential for evaluating the effectiveness of physical activity interventions that aim to maintain or improve older adults’ function. However, assessing motor competence in older adults who have difficulties walking or standing is challenging, because few instruments or guidelines are appropriate for these frail older adults. This article aims to describe challenges in evaluating motor function among frail older adults, discuss strategies for adapting motor function assessments to their home settings, and provide recommendations for future clinical trials so that older adults with ambulatory difficulties can benefit from motor function assessment and physical activity programs. Data came from the baseline assessment of 116 participants of an ongoing clinical trial, “Promoting Seniors’ Health with Home Care Aides (Pro-Home)”. Our results demonstrated that the Pro-Home study involved participants who would be typically excluded from clinical trials and that the two instruments selected or developed for Pro-Home (Short Physical Performance Battery, Pro-Home Ankle Range of Motion Measure) captured a wide range of lower extremity motor competence with no or few missing data. Recommendations for future studies include knowing the target population thoroughly, developing trust and rapport with all parties involved, and continuously collaborating with interviewers who conduct assessments.

## 1. Introduction

Motor function is essential for older adults to perform daily activities and remain in their home as they age. Physical activity helps maintain or improve motor function, and it is almost never too late to start or resume physical activity [1,2]. However, older adults with disabilities, especially those with ambulatory difficulties, have limited opportunities to participate in physical activity. Clinical trials rarely target older adults who have difficulties walking or standing up [3,4], and most existing motor function assessment instruments do not work well for frail older adults [5,6]. The overarching goal of this paper is to describe challenges in evaluating motor function among frail older adults, discuss strategies for adapting motor function assessments to their home settings, and provide recommendations for future studies on community-dwelling frail older adults. The data came from a larger study: “Promoting Seniors’ Health with Home Care Aides (Pro-Home): A Randomized Controlled Trial (RCT).” Pro-Home RCT is an ongoing study whose ultimate goal is to examine the effectiveness of a low-intensity physical activity program delivered by home care aides for their older clients in a publicly funded home care program in a large ethnically diverse city in the United States [7,8]. This current article intends to shed light on the process of assessing study participants’ motor function in their home settings, while highlighting the inclusivity of our randomized controlled trial that targets older adults with daily activity limitations. More specifically, this paper (1) describes the process of selecting and adapting measures of lower extremity motor function in community-dwelling older adults with daily activity difficulties such as walking or standing (Methods section), (2) demonstrates how our measurement strategies worked in their homes (Results section), and (3) provides recommendations for motor competency assessment in community-dwelling older adults who need assistance with daily activities (Discussion section).

## 2. Materials and Methods

### 2.1. Data

Data came from “Promoting Seniors’ Health with Home Care Aides: A Randomized Controlled Trial” (Pro-Home RCT). The analytic sample included 116 Pro-Home participants who completed baseline in-home assessment before 16 March 2020, when our project transitioned to remote operations due to the COVID-19 pandemic. Trained research interviewers visited participants’ homes three times at the baseline: (1) in-home screening/informed consent visit (study participants’ sociodemographic information, interviewers’ observation of assessment settings), (2) in-home main interview and assessment visit (typically 45–60 min), including motor function assessment and interviewers’ observation of interview/assessment, and (3) cognitive assessment visit. 

#### 2.1.1. Pro-Home Study Design 

Pro-Home’s primary goal is to determine whether a low-intensity physical activity program delivered by home care aides would preserve or improve the function of community-dwelling older persons who receive in-home services from the Illinois Department on Aging Community Care Program. Home care clients receiving services from our partner agencies were eligible for the Pro-Home study if they were aged 60+; had cognitive and physical function sufficient to follow directions and participate in our interviews and study activities; had a home care aide who was willing to participate in the study; and were fluent in English or Spanish. Detailed eligibility criteria are available elsewhere [8]. 

Upon completion of baseline assessment, participants were randomly assigned to a physical activity program or an active control group. Participants’ home care aides took part in a baseline program training session provided by the research team. Then, home care aides would deliver the program to their participants and encourage them to do the activities daily for 4 months. All participants were asked to complete two follow-up assessments at Month 4 and Month 8 (a two-group pre-test post-test design, with random assignment). A detailed research protocol is available elsewhere [8]. 

Pro-Home’s physical activity program is based on Healthy Moves for Aging Well^TM^, an evidence-based low-intensity physical activity program for community-dwelling older adults with daily activity difficulties developed by the Partners in Care Foundation [9]. Healthy Moves involves three easy-to-learn moves that were adapted from the Senior Fitness Tests [10]. One move targets upper-body function; the other two moves, Step-in-Place and Ankle Point and Flex, target lower extremity function. 

#### 2.1.2. Study Context and Participant Recruitment

The Pro-Home RCT involves older adults who receive in-home services from the Illinois Department on Aging Community Care Program. The Community Care Program covers persons aged 60 or older with limited assets (USD 17,500 or less) who need assistance with daily activities as determined by the Determination of Need assessment [11]. 

Participants were recruited through large in-home service providers in the Community Care Program. Research opportunities were announced in multiple ways, including brochures and letters circulated among home care aides distributed at home care branch offices, at in-service training sessions, and by home care agencies’ field coordinators who visited the homes of older home care users or by care coordinators who visited older service users to conduct care needs assessment. Home care users who provided written consent to release their contact information to the Pro-Home research team were contacted and screened by the research team. Some home care users initiated phone calls to the research team voluntarily, using the phone number listed in the recruitment materials. 

The Pro-Home RCT builds on the Pro-Home Pilot (a one-group pre-test post-test design), which provided the research team with opportunities to test and improve the feasibility of motor function assessment with our target study population [7].

### 2.2. Lower Extremity Functional Assessment 

#### 2.2.1. Selection Criteria

The Pro-Home study called for performance-based tests that would be (1) appropriate for the target population, (2) sensitive to change and responsive to the physical activity intervention to be tested, (3) practical to administer in the study context, and (4) valid and reliable psychometrically [12,13]. We used these inter-related criteria to identify, select, adapt, or develop measures specifically for the Pro-Home study.

- Appropriateness for the target population. We needed performance-based tests that would be feasible, safe, and acceptable for community-dwelling older adults who have difficulties with daily activities such as walking or standing up. Our preliminary and pilot studies provided our study team with hands-on experiences working with community-dwelling older adults who have difficulties with daily activities such as walking or standing up. Those studies also helped us understand our target population’s health and sociodemographic characteristics [7,14,15]. Those prior experiences helped us assess what would be feasible, safe, and acceptable for the study participants.
- Sensitivity to change and responsiveness. We needed measures that could detect a meaningful or clinically important change in lower extremity motor function within or across study participants, which could be expected of the low-intensity physical activity intervention and would be sensitive to change and responsive to the intervention in the target population [16,17]. If too easy or too difficult for the target population, performance tests could produce ceiling or floor effects, where large proportions of participants would score the minimum or maximum points possible for the performance test.
- Practicality of administration. We needed performance-based tests that would be practical for trained interviewers to administer for frail older adults in their home settings. Interviewers would need to bring whatever equipment were necessary for the tests from our research office into participants’ homes. We needed tests that (1) would minimize burden (e.g., time, exertion) on study participants and interviewers, (2) could be administered safely by the trained interviewer without formal clinical training (e.g., physical therapy training), (3) would allow participants to use assistive devices (e.g., cane, walker), if needed, in some of the lower extremity motor function tests (e.g., walking or gait speed test), (4) would not require large, heavy, or special equipment, and (5) would be relatively easy for the interviewer to score.
- Measurement validity and reliability. Our priority was to select performance tests that have been developed and validated for community-dwelling older adults with functional limitations. In particular, we needed an instrument that would validly and reliably measure lower extremity motor function targeted by two moves: Seated Step-in-Place and Ankle Point and Flex.

#### 2.2.2. Selected Instruments

We selected two instruments to measure lower extremity motor function: (1) the Short Physical Performance Battery (SPPB), a widely-used performance-based tool to assess older adults’ balance, gait speed, and lower extremity strength [18]; and (2) the Pro-Home Ankle Range of Motion Measure (PHAROMM), a measure of ankle flexibility developed by the Pro-Home team for older adults who have difficulties with daily activities such as walking or standing. 

##### 2.2.2.1. Short Physical Performance Battery

Selection Process

SPPB [18] was chosen as a measure of motor function targeted by one of the moves tested in the Pro-Home Study: Seated Step-in-Place. This move is designed to improve the strength and endurance associated with daily activity functions that involve the lower extremities, such as walking from room to room, getting to the toilet, shopping for groceries, and collecting the mail. 

SPPB, originally developed for community-dwelling older adults with different levels of function, is one of the most widely used measures of lower extremity motor function in older adults [6,18,19,20], including those with functional limitations [21]. It is practical to administer: SPPB allows for the use of an assistive device during the assessment. It requires little equipment or space, and can be administered in various contexts including home settings [13]. Training materials for assessors are publicly available [22]. It is an established measure that has evidence of reliability and validity [17,23,24,25] and reasonable responsiveness [16,17,23]. Furthermore, SPPB produces scores even when participants are not able to perform the test completely or the test is not attempted [18]. 

Among existing lower extremity motor function measures, SPPB stood out as an appropriate measure for the Pro-Home study. Most other performance-based lower extremity motor tests involve movement, like standing up and walking for a significant distance. Such tests would be unsafe or too difficult for the target population of home-bound older adults. For example, Senior Fitness Tests, which served as the basis of Healthy Moves, ask participants to practice walking performance tests (a 2 min Step-in-Place and 6 min walking test) before the test day. These tests would be too physically demanding for our target population with daily activity difficulties.

Adaptations

During pretests and pilot tests of the performance-based assessments, the Pro-Home team refined the assessment procedures. For example, the original SPPB involves three balance tests (side-by-side, semi-tandem, and tandem; see details in Measurement section), a gait speed test (available in two versions: 3 m or 4 m), and two chair stand tests (single chair stand and multiple chair stand). Due to space limitations in participants’ homes, we chose the 3 m version of gait speed tests. Participants were allowed to use an assistive device if needed. However, interviewers encouraged participants to walk without their devices if they felt safe to do so. We followed the original SPPB instructions to perform the tests with the following adaptations to ensure safety.

- Prior to each SPPB subtest, the interviewer demonstrated the subtest and asked the participant, “Do you feel safe doing the measure?” The test would be conducted only when participants felt safe doing it.
- Interviewers were trained to assess the appropriateness of participants’ footwear for SPPB subtests.
- For the chair stand subtest, interviewers were instructed to position the chair against the back of a wall, to prevent the movement of the chair.
- For the balance and gait speed subtests, interviewers were instructed to stand and walk close to the participants.

Measurement

We administered the original version of the SPPB tests among the participants following the adapted protocol. The SPPB tests were conducted after an interview session with the participant, which typically lasted for 30–40 min. Breaks and water were provided as needed. The score for each of the three components, balance, gait speed, and chair stand, ranged from 0–4, and the total SPPB score ranged from 0 to 12, by summing the three component scores. 

Specifically, for the balance tests, all participants started with the side-by-side test, the easiest of the three balance tests. They received a score of 1 if they could stand in a side-by-side position for 10 s, and proceeded to the semi-tandem test. Those who could not hold the position for 10 s or did not attempt it received a score of 0, and would not be administered the semi-tandem and tandem tests. For the semi-tandem test, participants received a score of 2 if they could stand with the side of the heel of one foot touching the big toe of the other foot for 10 s, and a score of 0 if they could not hold the position for 10 s or did not attempt it. Those with 0 points would not proceed to the tandem test. For the tandem test, participants received 2 points if they could stand with the heel of one foot in front of and touching the toes of the other foot for 10 s, 1 point if they held the position for 3–9.99 s, and 0 points if they held it for less than 3 s or did not attempt the test. A summary balance score was created by adding up the scores of the three balance tests.

The 3 m gait speed test was administered twice among participants, and the use of assistive devices (e.g., cane, walker) was allowed. The faster time of the two trials was used to assign a score: 0 points = unable to do the walk, 1 point = time > 6.52 s, 2 points = time 4.66–6.52 s, 3 points = time 3.62–4.65 s, and 4 points = time < 3.62 s.

For chair stand tests, participants started with the single chair stand. Participants who needed their arms to push off and stand up from the chair received a score of 0 for the chair stand component. Those who could stand without using their arms proceeded to the multiple chair stand test (stand up from a chair 5 times without using arms). The scores were determined as follows: 0 points = unable to complete 5 chair stands or completed stands in >60 s, 1 point = time 16.70–60 s, 2 points = time 13.70–16.69 s, 3 points = time 11.20–13.69 s, and 4 points = time 11.19 s or less. 

##### 2.2.2.2. PHAROMM

Selection Process

In contrast to the case of Seated Step-in-Place and SPPB, we were not able to identify performance-based tests of the function targeted by the Ankle Point and Flex move that would meet the selection criteria presented in Section 2.2.1. The Ankle Point and Flex move is intended to improve ankle flexibility, to increase the ability to lift toes to avoid tripping, and to increase blood circulation to manage ankle swelling. 

Existing measures of ankle flexibility, or ankle range of motion (AROM), either require large equipment (e.g., an examination table) or require the study participant to stand and lunge forward [26,27,28]. It quickly became clear that those existing measures would not be appropriate, safe, feasible, or practical for our Pro-Home research interviewers to use in assessing ankle flexibility in frail older adults in their home settings. The Pro-Home’s target population includes those who have difficulties walking or standing up on their own, so it would not be safe or feasible for all participants to lunge forward. Additionally, in-home assessment requires the research interviewer to carry all the assessment equipment and set it up in the participant’s home. As a result, we were unable to find ankle flexibility measures that were appropriate, safe, and practical for our target population.

Adaptations

The challenge of not being able to find an existing AROM measure led to an opportunity to develop our own instrument, the PHAROMM. During our pilot study [7], we developed an alternative AROM measure that used portable equipment (i.e., a folding goniometer that can fit into a clothing pocket, a folding stool, and a folding chair) and did not require the participant to stand or lunge. The development of the new AROM measure was an iterative process, where the research team collaborated closely with one of the study interviewers who knew how to use a goniometer from his rehabilitation job (C.O.; see the Acknowledgement section). During the pretest stage of the Pro-Home pilot project, the Principal Investigator (PI: N.M.) and her research assistant found an analog goniometer (typically used in rehabilitation settings) difficult to read in low light. Subsequently, the team found a goniometer with an electronic screen that displayed the degrees of ankle range of motion, and they developed the first version of the AROM measurement protocol. 

With the start of the Pro-Home randomized controlled trial, we revisited the AROM measurement protocol. At an early stage, we invited the experienced interviewer (C.O.) from the pilot stage of Pro-Home to share his experience of administering the AROM with the research team. In collaboration with this experienced interviewer (C.O.) and the research interviewers, the Pro-Home research team reviewed additional literature on AROM measures, refined the measurement protocol, and pretested it as part of the initial interviewer training sessions. The refinements included (1) adding a script for the interviewer to use during the AROM measurement, (2) enhancing instructions on how to position the participant’s body in the chair with one leg propped on the edge of the footstool and how to place the goniometer with respect to visual images (i.e., the goniometer in relation to anatomical landmarks) to guide the interviewer, (3) asking participants to point and flex the foot twice to warm up, and (4) checking participants’ perceived difficulty level with the pointing and flexing of each foot.

Measurement

Trained with the refined protocol for PHAROMM, the interviewer performed the AROM measurement as follows. The interviewer first prepared the participant for the AROM measurement process, saying “We are going to measure the flexibility of your ankles”. The interviewer asked the participant to remove their footwear and sit in an upright position, supported by the back of the chair. The interviewer unfolded the foot stool and placed one of the participant’s feet on the far edge of the stool. The interviewer moved the stool until the femur was parallel to the floor and the bottom of the thigh touched the seat of the chair. The interviewer ensured the hip, knee, and foot aligned in the sagittal plane. 

As a warm-up, the participant was instructed to move their foot up and down (dorsiflexion and plantarflexion) twice, with the right ankle. Measurement with the goniometer was not carried out during the warmup. The participant was asked to rate how difficult it was to move their ankle through the range of motion on a Likert scale, from very easy (1) to very difficult (5). Then, the interviewer placed the goniometer on the lateral aspect of the ankle with the goniometer’s fulcrum immediately below the lateral malleolus. Participants were not asked to position their ankle at neutral (i.e., 90° in the sagittal plane) before measurement, since not all older adults can move their ankle into that position. The interviewer then asked the participant to flex their ankle by pointing their foot toward their body as far as they could (the interviewer gently touched their foot to cue, as needed). As the participant ranged their ankle, the interviewer moved one of the goniometer arms to align with the participant’s fifth metatarsal, and read the degrees displayed on the screen. The participant was instructed to point their foot away from their body as far as they could (“like pushing on a gas pedal”). Again, the interviewer moved the goniometer arm and read the degrees. Measurements were collected and recorded for both ankles. 

A final PHAROMM score was derived by calculating the combination of dorsiflexion and plantarflexion of each ankle separately.

### 2.3. Interviewers 

Interviewers play a critical role in motor function assessment. Pro-Home interviewers worked closely and developed a rapport with the participants, to make the measurements work. Interviewers also reported any issues that they observed or experienced to the office-based research team, to address those issues. This process would determine the quality of data. The following sections discuss our approaches to working with interviewers to implement motor function assessment in five areas, including (1) interviewer hiring, (2) training, (3) measurement protocol development, (4) continued communication, and (5) tools to enhance interviewers’ data collection experience.

#### 2.3.1. Hiring

Recruiting qualified, committed research interviewers is perhaps the most important factor that determines the success of motor function assessment. We targeted the recruitment toward people with a background and/or a degree in public health or related discipline (e.g., kinesiology, nursing, occupational therapy, physical therapy, medicine, sociology, psychology, social work). Qualifications included experience working in community-based research programs, ideally with people aging with disabilities, and/or in a clinical setting. Given that Pro-Home study participants were either English or Spanish speaking, we recruited mostly bilingual interviewers. To establish trust and rapport with study participants, we specifically emphasized excellent interpersonal and communication skills as critically important qualifications. Our interviewer team was composed of five bilingual members and one English-speaking member, each with strong skillsets and qualifications for this role. The number of interviewers hired was determined based on the geographic area we recruited from. We updated and retrained the core interviewers throughout the data collection period. 

#### 2.3.2. Training

Interviewer training was guided by the following plans and principles, as shown below.

- Curriculum. The interviewer training curriculum, led by the research team staff, was comprised of 30 h hands-on in-person training during the first 4 weeks. It covered topics including communicating with older adults, safety, protection of human subjects, data collection, performance-based assessments, use of technology, and role plays. New interviewers shadowed experienced interviewers and conducted assessments under supervision, prior to being certified to perform these tasks independently.
- Individualized training. Each interviewer had unique strengths. Some excelled in health literacy, and others excelled in motivating and communicating with older adults, the use of electronic devices, or addressing data quality. We identified each interviewers’ strengths and areas of growth to personalize additional training and practice.
- Co-learning. A highly experienced interviewer who worked in our pilot project that developed the AROM measurement protocol helped train the new interviewers. We intentionally recruited interviewers with complementary strengths, so that interviewers would learn from each other through role playing and discussion. For example, interviewers with clinical backgrounds helped others to correctly identify anatomical landmarks. Those who administered SPPB in other studies or had experience communicating with older adults shared their experience with other team members. Once the project matured, then we could identify a lead interviewer who could carry out certain training components independently.
- Bilingual interviewer training. Interviewers completed the English curriculum described above. Once certified in English, they completed additional practice assessments, and shadowed assessments in Spanish with a bilingual lead interviewer or research staff.
- Refresher training. We provided refresher training every 6 months during the course of the study. Refresher training varied in length, based on the topic (1 to 3 h). When our project transitioned to remote operations due to the COVID-19 pandemic, these trainings focused primarily on added administrative tasks (i.e., scanning, network set up and access, etc.).
- Continuous improvement and refinement of training materials. Updating training materials was especially important for the AROM measurement we developed. To standardize assessment procedures, we wrote and refined scripts and created training videos of the assessments being carried out.
- Ensuring competency and consistency. At the beginning of the project, the interviewer trainer (a member of the research staff) would observe interviewers administering assessments to other staff and to study participants in the home. Later on, after a lead interviewer was identified, the lead interviewer would observe other interviewers and coach them, as needed, during refresher training sessions or in study participants’ homes. We also conducted a training session in which interviewers, including the lead interviewer, measured the AROM of mock participants, and received feedback based upon the results.

#### 2.3.3. Protocol

We enhanced the assessment protocol documents developed during the Pro-Home Pilot, to ensure measurement consistency and safety for both participants and interviewers/assessors in the current Pro-Home RCT. The motor function assessment protocol describes the purpose of each measure, equipment needed, procedures with visuals, scripts for interviewers to communicate with the participants, scoring rules, and additional special instructions (please see Section 2.2.2.1 and Section 2.2.2.2 for protocol development and adaptation). We developed protocol documents iteratively and collaboratively. If an interviewer encountered a situation and had questions, we discussed it and clarified the protocol, if necessary. 

The protocols were made available to interviewers via paper copies in their individual research interview binder and electronic copies saved on a secure university network. Interviewers were instructed to always bring their binders with a copy of the protocol during each home visit. The binder also included hard copies of all consent forms in English and Spanish, a calendar, scoring sheets, and copies of incentive receipts. 

#### 2.3.4. Continued Communication

For motor assessment to be carried out effectively and safely, communication among the research staff, interviewers, the participant, the participant’s family, and the home care aide was critical. Interviewers were instructed to text the office research team upon arrival at the participants’ home and after completion of the study visit. If at any point during an assessment there was a question, interviewers reached out to the office research team for support or to address their questions. Participants were provided with a magnet with the study number and were instructed to reach out to the office research team with any questions, rather than to their interviewer. 

The Pro-Home research team held monthly conference calls with interviewers to provide on-going training and updates, complementing the in-person refresher training sessions that were held twice a year. At the monthly meetings, interviewers shared their experiences during participant visits so that fellow interviewers could learn how to handle similar experiences, should they occur. The identified lead interviewer was a liaison between the interviewer group and the research team.

#### 2.3.5. Tools to Enhance Data Collection Experience

Interviewers were provided with a backpack that was essential to carry all their equipment for their in-home visits. The backpack included the binder, an iPad (for data entry), a wireless keyboard, folding stool (for PHAROMM), digital goniometer, portable charger and cables for the electronics, batteries, a stopwatch, measuring tape and blue painter’s tape (for SPPB), disinfecting wipes to clean the equipment after each use, lens wipes for the iPad, and hand sanitizer. Interviewers also brought water bottles for the participants and themselves, for during the assessments. Having all the equipment in a central place allowed interviewers to better transport other equipment such as the folding table and chair, which were important when addressing the challenges of in-home assessment with older study participants.

Interviewers’ observation of the neighborhood and home environment during the first home visit to screen/obtain informed consent allowed the interviewer and the study team to make accommodations so that the baseline visit of motor assessment was feasible. For example, the team sent a second interviewer to accompany the visit if the interviewer did not feel safe in the neighborhood. If the interviewer did not find space in the participant’s home appropriate for the motor function assessment, they would identify a space available outside the home (e.g., a hallway in the apartment building) during the screening visit, to be prepared for the baseline assessment visit. The interviewer also brought a folding chair or a portable table, as needed, for the baseline assessment. 

We built our data capturing and monitoring system in REDCap [29], a secure web platform that features real-time data collection, data entry and management (Developer: Vanderbilt University, Nashville, Tennessee, United States; University of Illinois Chicago REDCap Website: https://www.redcap.ihrp.uic.edu). REDCap streamlined the data capturing and monitoring process for our study. Interviewers used our study REDCap forms to record participants’ responses and document their field observations on participants and visits. Once the data collection and data entry were completed for a visit, a member of the research staff would review the forms to ensure the completion of the data and communicate with the interviewer about any issues. After the baseline assessment, the participant was randomly assigned to the experimental or control group in REDCap’s randomization module. REDCap was set up so that interviewers were blinded from knowing the participant’s group assignment, to minimize assessment bias during the follow-up visits. 

Interviewers multitasked during the assessment, which included reading instructions, demonstrating movement, timing the test, counting, and watching for participants’ safety. To ease the interviewer’s burden, we used paper forms to record the data of lower-extremity-function assessment. The data would then be manually entered into the study project in REDCap. Events or health conditions that might affect participants’ performance were also documented. Upon the completion of data entry, interviewers first self-checked the assessment data for obvious missing or unexpected values, and a designated research assistant re-checked the data to ensure accuracy. An open field on the REDCap form was used to document the data checking process. The data checking process helped the interviewers and the research team identify issues in the assessment and address them in a timely manner.

### 2.4. Other Measures and Analytic Strategy

#### 2.4.1. Participants’ Characteristics

##### Sociodemographic Characteristics

Study participants’ sociodemographic information was collected at the interviewer’s first visit to the participant’s home. The interviewer started with screening the participant for study eligibility. Then, after obtaining informed consent, the interviewer collected sociodemographic information, including age, gender, race, ethnicity, education, marital status, living arrangements, and equipment (i.e., assistive device) use in daily activities.

##### Motor Function Assessment

Study participants’ motor function was assessed during the interviewer’s second baseline visit (the main interview and assessment). The interviewer first collected self-reported measures of health and psychosocial characteristics directly from the study participant, and then moved onto the performance-based assessment, including the two lower extremity measures: the Short Physical Performance Battery [18] and the PHAROMM. A detailed description of the motor function assessment, the focus of this paper, is described in Section 2.2.

##### Cognitive Function Assessment

Cognitive function was assessed using an iPad with the NIH Toolbox Cognition Battery (NIHTB-CB) application (app) installed [30], a reliable and valid tool for older adults with or without health issues [31,32,33,34]. Interviewers were trained by neuropsychologists (W.S., S.L.; see the Acknowledgement section) on the team to administer NIHTB-CB. The 5 tests used were the Flanker Inhibitory Control and Attention Test, List Sorting Working Memory Test, Dimensional Change Card Sort Test, Pattern Comparison Processing Speed Test, and Picture Sequence Memory Test. The NIHTB-CB app automatically provides age-corrected standard scores for participants aged 85 or younger when they have completed the test. The age-corrected standard scores were not available for participants aged above 85.

#### 2.4.2. Interviewer Observations

After each interview or assessment visit, interviewers provided their observations about the participant, the interview or assessment, and its environment.

##### Perceived Neighborhood and Home Environment

When the interviewer visited the participant’s home for the first time to screen the participant, the interviewer was asked to observe their neighborhood and home environment and select the response category that was closest to their perception (e.g., how safe the neighborhood was, and whether the home environment would be appropriate for motor function assessment in terms of cleanliness, space, safety, and the availability of a chair and table required for the motor function assessment).

##### Perceived Interview Quality

The interviewer rated their interview experience (e.g., perceived interview quality, the presence of others during the visit) and the study participant’s levels of comprehension, enjoyment, tiredness, memory, hearing limitations, and strain as perceived by the interviewer, by selecting the response category that was closest to their perception.

#### 2.4.3. Analytic Strategy

Descriptive statistics of the data were produced by using Stata 17.0 [35]. The study was approved by the University of Illinois Chicago Institutional Review Board (Protocol #: 2016-0689).

## 3. Results

### 3.1. Sample Characteristics

As recipients of publicly funded home care services, Pro-Home study participants were older adults with low socioeconomic status and limitations in both physical and cognitive functions (Table 1).

Participants were 76 years old on average (SD 8.7, range 61–96), typically female (74.1%), unmarried (75.0%), and lived alone (46.4%). Most participants were Black/African American (50.9%) or Hispanic (32.8%). Close to 4% had no formal education, and almost one half of the participants did not attain the high school level of education.

Participants had significant functional limitations. A significant proportion of participants reported the use of assistive devices: two-thirds used equipment (e.g., cane, walker, etc.) to cross a room, and one-third used equipment to get in or out of bed. More than half of the participants had 5+ health conditions at the time of the baseline assessment. More than half reported the following conditions: high blood pressure or hypertension (81.9%), arthritis or rheumatism (81.0%), high cholesterol (55.2%), and vision problems such as glaucoma or cataracts (50.0%).

More than one-half of the study participants showed signs of cognitive impairment, although the Pro-Home study included only those who could follow directions and participate in the study activities, as mentioned earlier. Participants were eligible to participate in the study only if they passed the six-item screener [36] and three selected tests (3-stage command, forward and backward number counting, and body part naming tests) from the Modified Mini-Mental State Examination [37]. Even though the participants passed the above-mentioned cognitive screening tests, more than half of the participants had NIH Toolbox Cognition Battery scores that are typically considered as signs of cognitive impairment in one or more tests. Specifically, 67 persons (57.8%) scored below 1.5 SD from the mean of the age-corrected standard score in one or more tests (scored below 1.5 SD in 1 or 2 tests, N = 48; in 3 or 4 tests, N = 19; in 5 tests, N = 0). Scoring below 1.5 SD from the mean of the age-corrected standard score is a criterion of mild cognitive impairment commonly used by other researchers [38]. Furthermore, 15 persons (12.9%) were unable to complete at least 1 of the 5 tests in the NIH Toolbox Cognitive Battery. Specifically, 14 participants started the test but failed during the practice round and were not allowed to participate in the actual test (9 failed the practice on 1 test, and 5 on 2 tests). One person could not participate in any of the tests, due to vision loss.

**Table 1:** Baseline participant characteristics (N = 116) ^1^.

Demographic and Health Variables	Mean or N (%)
Age	76 (SD = 8.7; Range 61–96)
Female	86 (74.1)
Race/Ethnicity	
Black/African American	59 (50.9)
Hispanic ^2^	38 (32.8)
Non-Hispanic White	16 (13.8)
Two or more races	3 (2.6)
Education	
No formal education	4 (3.5)
Less than high school	50 (43.1)
High school graduate ^3^	16 (13.8)
Some college	31 (26.7)
College graduate or above	12 (10.3)
Not married currently	87 (75.0)
Live alone	54 (46.6)
Equipment use in daily activities	
Use equipment to cross a room	73 (64.0)
Use equipment to get in/out of bed	36 (31.3)
Chronic conditions ^4^	
>5 health conditions	62 (53.4)
Cognition ^5^	
Scored below 1.5 SD from the SS ^6^ mean in:	
At least 1 test ^6^	67 (57.8)
1 test	29 (25.0)
2 tests	19 (16.4)
3 tests	9 (7.8)
4 tests	10 (8.6)
5 tests	0 (0)
Unable to complete at least 1 test ^7^	15 (12.9)

^1^ A total of 117 participants completed in-person baseline assessment before 16 March 2020. Of these, one person was a screen failure that we failed to screen out (not meeting the inclusion criteria at the time of screening) and thus was not included in the analysis. ^2^ Of the 38 Hispanic participants, 33 were Hispanic White, 1 was Hispanic Black, and 4 selected Other Race when responding to the race question (2 said Hispanic and 2 said Mexican). ^3^ Of the 16 high school graduates, 11 obtained a high school diploma, 4 obtained a high school equivalency certificate (General Educational Development, or GED), and 1 did not obtain either. ^4^ A list of 23 chronic conditions adapted from Charlson Comorbidity Index [39]. More than half reported the following conditions: 95, high blood pressure or hypertension (81.9%); 94, arthritis or rheumatism (81.0%); 64, high cholesterol (55.2); and 58, vision problems such as glaucoma and cataract (50.0%). ^5^ The five tests we used in the battery were: Flanker Inhibitory Control and Attention Test, List Sorting Working Memory Test, Dimensional Change Card Sort Test, Pattern Comparison Processing Speed Test, and Picture Sequence Memory Test. ^6^ SD = Standard Deviation; SS =Age-corrected Standard Score. ^7^ Unable to complete (1) 1 test: N = 9; (2) 2 tests: N = 5; (3) 5 tests: N = 1.

With the sample characteristics described above, how well did we capture the lower extremity motor function at baseline? The next two Section 3.2 and Section 3.3 answer this question by presenting descriptive statistics of SPPB and PHAROMM respectively.

### 3.2. Short Physical Performance Battery

SPPB produced no missing data, despite the fact that 47% of our participants attempted all the SPPB tests, as shown in Table 2. Most participants attempted (felt safe to do) the side-by-side test, the easiest of the three balance tests, and the gait speed test. Specifically, 109 participants (94%) completed the 3 m walk, and of these, 37 (33.9%) used an assistive device during the walk (e.g., cane, walker), suggesting the importance of allowing assistive device use in the test. Close to two-thirds attempted the chair stand tests.

**Figure 1:**
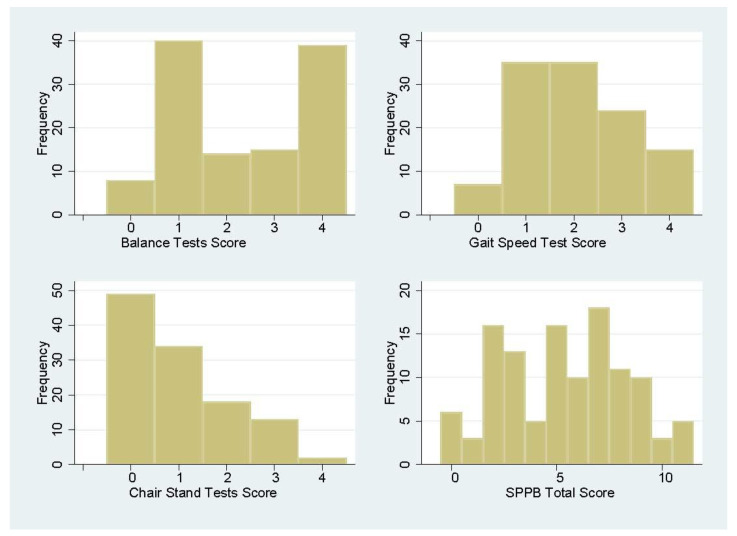
Short Physical Performance Battery (SPPB) score distributions.

The SPPB scores captured different levels of participants’ lower extremity function. Only a small proportion of participants scored at a maximal or minimal level, suggesting a low risk of having a ceiling or flooring effect. As shown in Table 2 and Figure 1, no participants scored the maximum possible total SPPB points (score of 12), and only 5% scored 0 of all SPPB tests. The majority were able to score at least 1 point from balance tests or the gait speed test, and a small proportion of the participants scored full points from the tests. In contrast, about 40% of the participants scored 0 on chair stand tests.

### 3.3. PHAROMM

All but one participant felt safe doing the PHAROMM, indicating the appropriateness of the instrument. The participant who felt unsafe to participate in the assessment had pain and health issues in the right leg, and thus proceeded with the test with the left ankle only. Of those who felt safe doing the assessment, one participant experienced difficulty and pain during the warm-ups because of leg surgery, and therefore interviewers discontinued testing, due to safety concerns.

**Figure 2:**
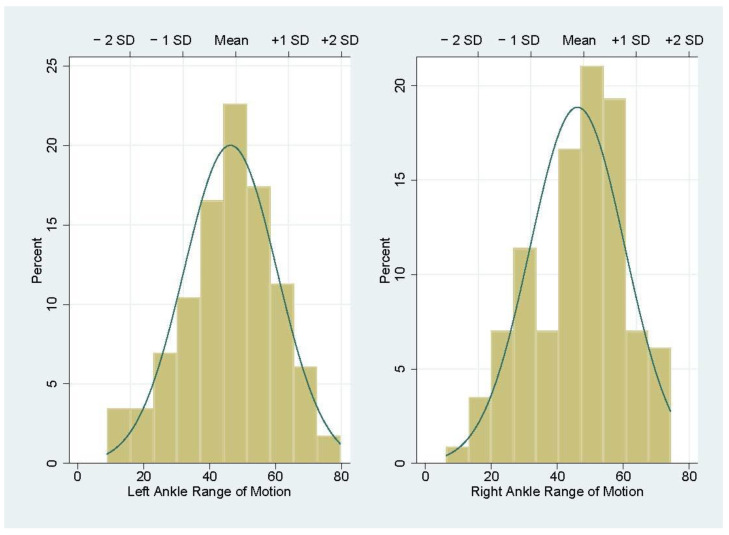
Pro-Home Ankle Range of Motion Measure (PHAROMM) score distribution.

On average, participants had 48 degrees of range of motion in both ankles (SD = 16 for both ankles), ranging from 9 to 80 degrees in the left ankle and 6 to 74 degrees in the right ankle. The distribution of the PHAROMM data was close to normal (Figure 2).

### 3.4. Post-Visit Interviewer Perception

This section addresses the importance of using post-visit interviewer observation forms to communicate with the team about the context of the assessment and make accommodations as needed. Interviewers were asked to rate their perception of neighborhood safety and home environment upon the completion of their initial in-home visit (screening), and their perception of the visit and participants after completing the baseline assessment. Our results showed that all interviewers completed their observation ratings upon the completion of each visit separately, and that it is practical to administer the selected lower extremity motor function instruments among Pro-Home participants in their homes.

Interviewers’ observations on participants’ neighborhood safety and home environment provided valuable information for the team to work with, with the interviewers, to make the necessary adjustment for the following baseline assessment. Interviewers felt participants’ home neighborhoods were safe, except for two cases (Table 3). A little more than 10% of the participants’ homes were untidy or overcrowded with furniture or objects, leaving little space for motor function assessment. Close to one-third of the participants did not have an appropriate chair for SPPB (i.e., a sturdy chair without wheels), and close to one fourth of the participants did not have a table that the interviewer needed during the assessment. The lighting conditions were adequate for performing the baseline assessment for all but one participant. Overall, interviewers did not feel a second interviewer needed to be present for participants’ or interviewers’ safety reasons to perform the baseline assessment in most participants’ homes.

Regarding the rating of the baseline assessment experience, interviewers believed that the quality of most sessions was high (64.7%) or adequate (31.9%), as shown in Table 4. About two thirds of the participants had other people present during the assessment visit, such as family members, their home care aide, or a health care professional. Interviewers did not experience barriers from obtaining data during the visit except for one case. Interviewers also provided their perceptions of the participant’s level of comprehension, enjoyment, tiredness, memory, hearing limitations, and strain during the baseline assessment. Overall, interviewers perceived that most participants had a good or excellent understanding of the questions asked (92.2%) and seemed to have enjoyed the assessment session (99.1%). However, interviewers perceived that a significant proportion of participants looked tired (52.6%), had difficulty remembering questions (37.9%), or had difficulty hearing what interviewers said (21.7%). Despite these challenges, interviewers reported that only a small portion of participants required frequent repetition of questions (12.1%), appeared strained by leaning forward and/or watching the interviewer’s lips (3.5%), or failed to react to the interviewer’s questions without watching the interviewer’s lips (1.7%).

## 4. Discussion

### 4.1. Pro-Home Lower Extremity Motor Assessment

The Pro-Home study provided a rare opportunity to make motor function assessment inclusive of frail older adults who have difficulties with daily activities, such as walking or standing. This article demonstrates that despite challenges in motor function assessment among frail older adults, Pro-Home participants felt safe and attempted most tests from the study’s lower extremity function assessment, which included the SPPB and PHAROMM. The scores captured a range of functional abilities of the participants. Interviewers generally reported positive experiences from the assessment visits.

Pro-Home participants tended to have low socioeconomic status, multiple health conditions, and physical and cognitive limitations, which posed challenges to in-home motor function assessment. Not unexpectedly, because our participants were recruited from a publicly funded Medicaid waiver program for those with long-term care needs, they appeared to score lower in all components of the SPPB compared to previous studies that administered SPPB among community-dwelling older adults [19,24,40]. In fact, our participants had similar scoring patterns to studies with hospitalized geriatric patients [41,42]. Understandably, participants typically felt safe to perform the assessments that were easy for them, given their functional limitations (e.g., the side-by-side test was the easiest balance test, and most attempted it). Assessments were made further accessible because we allowed the use of an assistive device (e.g., using a cane to walk during gait speed test). The variations in test difficulty and the allowance of assistive device use in SPPB produced a range of SPPB scores that can characterize our participants’ lower extremity function. In summary, with adaptations, SPPB is an appropriate and safe tool to measure lower extremity function among community-dwelling frail older adults in their homes.

We found that the average combined AROM among participants in our study was 48° in either ankle. Studies have indicated that the combined AROM in the general population is between 65° and 75° [43]. We expected that the frail older participants in our study would have less AROM than adults in the general population, and this expectation bore out. The average combined AROM we found is much lower than the average AROM of non-frail community-dwelling older women in two studies that used measures which also required the participants to dorsiflex and plantarflex with little assistance [44,45]. In these two studies, the means of combined ankle AROM ranged from 68 to 75°. The differences were not surprising, given that our study participants had difficulties with daily activities, including walking and standing. Differences in protocols and samples may also explain the differences in averages. For instance, we had participants warm up their ankles, while the protocol used in Jung and Yamasaki’s study [44] did not include a warm-up; active warm-ups are associated with increased ankle range of motion [46].

Pro-Home’s continuous training, appropriate protocols, communication strategy, and tools (e.g., backpack, REDCap) to enhance data collection experience have equipped our interviewers with the skills, knowledge, and resources to carry out motor function assessment, and made them feel comfortable going into the communities of participants who are usually underrepresented in research in terms of race, ethnicity, socioeconomic status and language. Interviewers generally reported positive perceptions of the participants’ neighborhood and home environment and of the participants themselves. Their ratings on neighborhood safety and home environment (e.g., space and equipment availability for assessment) prior to the baseline assessment was critical for the research team for understanding the context of the assessment and accommodating participants’ needs. As a result, most participants seemed to enjoy and understand the baseline assessment.

Both SPPB and AROM assess lower extremity motor function and have been demonstrated to predict health outcomes such as all-cause mortality [47], mobility [48], and falls [49]. However, assessing frail older adults’ lower extremity motor function is inherently challenging because (1) they have physical and often cognitive limitations; and (2), given the participants’ health and functional status, it was not practical to have study participants travel to a research laboratory under the control of the research team. Despite the lack of guidelines for measuring motor function in this population, general measurement selection criteria in healthy older adults, such as appropriateness, practicality, and psychometric properties (validity, reliability, and responsiveness), still apply [6,13]. Our study results highlight the importance of developing appropriate procedures to select and adapt instruments to accommodate community-dwelling frail older adults, and recruit and train qualified interviewers, which make it possible to safely assess motor function among older adults with functional limitations, including those who have difficulties with walking or standing.

### 4.2. Recommendations

In anticipation of challenges in assessing motor function in frail older adults who have difficulties walking or standing, Pro-Home developed and refined strategies for addressing those challenges. First, to address the lack of guidelines for selecting motor function measures appropriate for frail older adults, we defined criteria for selecting measures, learned how other researchers addressed those challenges by conducting literature review, and developed and refined plans for training interviewers. Second, to address the challenge of assessing frail older adults with unique sets of physical and cognitive challenges, we developed and adapted measurement protocols so that we can accommodate the needs of study participants and interviewers accordingly. Third, to address the challenge of conducting assessments in the study participant’s home setting that is not under the control of our research team, we had interviewers assess the home environment prior to the main assessment visit. We also prepared a standard set of equipment for assessment visits to facilitate interviewers’ tasks. Fourth, to address the anticipated variations in study participants’ function and home environment, we identified the key skills needed of interviewers, provided interviewers continuous training throughout the study, stayed in communication with interviewers during their home visits, and paid interviewers by the hours spent on each case rather than by the number of cases. Table 5 summarizes these anticipated challenges and Pro-Home strategies to address those challenges. 

Based on our experiences, we have developed 8 recommendations for future efforts to assess frail older adults’ motor function in their home settings as shown below and in Table 5.

1. Know your target population. Select measures appropriate for the target population, and adapt them as needed. Select measures that are more inclusive than others. For example, the SPPB gait speed subtest allows participants to use devices (i.e., cane, rolling walker, walker, rollator) which make the instrument inclusive of those who use assistive devices. Additionally, the SPPB balance tests consist of three tests that vary in the level of difficulty, so that the majority could complete at least one of the tests.
2. Do not skip pretests and pilot testing. Pretesting the assessment protocol within the research team and with collaborators helps identify and address potential problems before administering to the study participants.
3. Provide clear instructions and have interviewers demonstrate assessment procedures to study participants. Acknowledge that participants have different levels of educational background, health status, and cognitive or sensory function. Use a prepared script as a guide, so that interviewers can better explain and demonstrate each test. This process will help participants better understand the test procedures.
4. Know the home environment in advance. Homes have different physical features (e.g., flooring, lighting, furniture, and open floor space), which may be potential safety hazards. Pro-Home interviewers used the first screening visit to assess and document the availability of space and equipment, and identified safety issues. This step helped interviewers plan for the baseline lower extremity motor function tests (e.g., identifying space for the 3 m gait speed test in the home setting, assessing the need for bringing a folding chair from the research office).
5. Ask participants about their perceived safety prior to each test. Participants’ safety is a priority. An assessment should not proceed unless participants feel safe participating in it.
6. Train interviewers thoroughly and continuously. Comprehensive, hands-on initial and refresher training is essential. Detailed training reveals each interviewer’s skills, strengths, and areas of growth, thus facilitating individual training plans. Having one experienced, lead interviewer train others (or newly hired interviewers) is effective and efficient.
7. Develop a learning community with interviewers, office staff, students, and a senior collaborative team. Interviewers have direct contact with study participants. Valuing interviewers’ input and promoting collaboration among interviewers, office staff, and research team are critical elements for success.
8. Value interviewers’ contributions and incentivize appropriately. Participants may have a wide range of motor competence, and the time spent on in-home assessment may vary, depending on participants’ health, state of mood, and the context of the assessment visit. In rare cases, interviewers may need to split an assessment session into different days. Pay interviewers by the number of hours spent on a participant instead of by case. To keep interviewers engaged throughout the studies, it is important to incentivize interviewers appropriately.

### 4.3. Strengths, Limitations and Future Directions

Our study is one of the few that address practical guidelines needed for assessing lower extremity function in a special population, older adults with daily activity limitations, including those with ambulatory disabilities and long-term care needs. We share how we selected measures, the implementation of strategies, and our initial findings. We provide resources for other researchers and encourage them to involve community-dwelling frail older adults in clinical research, including, but not limited to, assessing motor competency.

We adapted the SPPB protocol to make it appropriate for our participants. SPPB has been widely validated and administered in older adults across different settings, and it is beyond the scope of this article to present the psychometric properties of the SPPB in our sample. 

We developed the PHAROMM. PHAROMM has face validity measuring ankle flexibility in older adults with daily activities difficulties (e.g., difficulties walking or standing up). At this initial stage of PHAROMM development, our efforts focused on making the PHAROMM feasible for and acceptable to study participants as well as for interviewers. This effort would in turn enhance the validity and reliability of the measurement. PHAROMM needs additional research to assess its validity, reliability, sensitivity to change, and responsiveness.

We relied on interviewers’ subjective ratings of the home environment and participants’ reactions to the assessment so that we can address issues identified by interviewers and support them as needed. Interviewers’ ratings may not be interpreted as objective assessment of the home environment. 

Overall, our strategies worked well for in-person assessment. Unfortunately, due to the COVID-19 pandemic, we had to discontinue in-person assessment during the study. A major area for future research is the development of remote motor function assessment in frail older adults who not only have physical functional limitations but also cognitive impairment. Physical functional limitations tend to coexist with cognitive impairment. Although the pandemic accelerated research on remote assessment of motor function (e.g., using consumer-focused wearables such as Fitbit), such remote assessment measures may not work well with frail older adults with ambulatory difficulties, because of their atypical movement patterns and use of ambulatory devices. Additionally, common wearables currently on the market do not sufficiently detect and distinguish some movements (e.g., detecting ankle movement) and the raw data that consumer-focused wearable devices collect are not available without special access provided by the manufacturer.

## 5. Conclusions

It is almost never too late to start or resume physical activity. “Keep moving” is a key message for people who are aging with or into disabilities. However, few evidence-based physical activity programs are appropriate for frail older adults who have difficulties walking and standing, and need daily assistance. Producing evidence of physical activities appropriate for this subpopulation of older adults requires safe and effective measures of motor function. Efforts should identify existing measures appropriate for the target population with favorable psychometric properties and, if needed, adapt the selected measures to the unique characteristics of frail older adults. Our project selected and adapted a widely used and validated assessment tool for lower extremity motor function (gait speed, balance, and strength), and we also developed a new measure to fill the gap in measuring ankle flexibility in frail older adults. We adapted our measurement protocols to different participants and their home settings, while ensuring consistency. We continuously trained interviewers and promoted collaboration among interviewers and internal and external stakeholders to continuously improve data collection and data checking procedures. The instruments we adapted and administered, and the strategies we adopted, captured a range of lower extremity motor functions consistent with our participants’ characteristics. Scientifically rigorous and practical motor function assessment is critical for providing evidence for programs that promote the function and wellbeing of the growing number of frail older adults dwelling in the community. 

## Figures and Tables

**Table 2:** Short Physical Performance Battery (SPPB): The rates of attempts and scores (N = 116).

Short Physical Performance Battery	Mean or N (%)
Missing Data	0 (0)
Attempted Short Physical Performance Battery	
All tests	55 (47.4)
Balance: Side-by-side test	108 (93.1)
Semi-tandem test	85 (73.3)
Tandem test	67 (57.8)
Gait speed test (3 m walk) ^1^	109 (94.0)
Chair stand: Single stand	75 (64.6)
Repeated chair stand	70 (60.3)
Short Physical Performance Battery Scores	
Balance	2.3 (SD = 1.4; Range 0–4)
Gait speed	2.0 (SD = 1.1; Range 0–4)
Chair stand	1.0 (SD = 1.1; Range 0–4)
Total	5.4 (SD = 2.9; Range 0–11)

^1^ Of 109 who attempted gait speed test, 37 (33.9%) used a walking aid to complete the tests.

**Table 3:** Neighborhood environment and home settings reported by interviewers (N = 116).

Neighborhood Environment and Home Settings	N (%)
Neighborhood safety	
Very safe	77 (67.0)
Somewhat safe	36 (31.3)
Not at all safe	2 (1.7)
Home settings	
Not clean	19 (17.4)
Overcrowded with furniture or objects	13 (11.2)
No space for 3 m gait speed test	5 (4.3)
No chair available for physical performance tests	35 (30.2)
No table accessible for assessments	27 (23.3)
Lighting conditions inadequate for assessments	1 (0.9)
Need for a second interviewer for baseline	10 (8.6)

**Table 4:** Interviewer baseline assessment experience and their perceptions of participants (N = 116).

Interviewer Observations	N (%)
Baseline assessment experience	
High or adequate quality	112 (96.6)
Presence of others (non-participants) during visit	76 (65.5)
Interviewers’ perception of participants	
Good or excellent understanding of questions	107 (92.2)
Enjoyed the baseline assessment session ^1^	115 (99.1)
Assessment seemed tiring for participants (a little to very ^2^)	61 (53.6)
Difficulty remembering questions (a little to some ^3^)	44 (37.9)
Difficulty hearing (a little to a lot ^4^)	24 (21.7)
Required frequent repetition of questions	14 (12.1)
Appeared strained by leaning forward and/or watching interviewer’s lips very carefully	4 (3.5)
Failed to react to interviewer’s questions and comments, if not watching interviewer’s lips	2 (1.7)

^1^ Enjoyment level–A great deal: N = 54; Quite a bit: N = 45; Some: N = 16. ^2^ A little tiring: N = 54; Very tiring: N = 7. ^3^ A little difficulty: N = 36; Some difficulty: N = 8. ^4^ A little difficulty: N = 17; Some difficulty: N = 6; A lot of difficulty: N = 1.

**Table 5:** Assessing motor function in frail older adults: Anticipated challenges, Pro-Home strategies, and recommendations.

Anticipated Challenges	Pro-Home Strategies	Recommendations
There are few guidelines for selecting motor function measures appropriate for frail older adults who have difficulties walking or standing.	Define selection criteria. Conduct literature review. Train interviewers. Pretest and pilot measurement protocols.	Know your target population. Do not skip pretests and pilot testing.
Administering motor function assessment among frail older adults is challenging, because they tend to have physical and cognitive limitations.	Develop and adapt protocol. Accommodate participants’ needs. Train interviewers.	Provide clear instructions and have interviewers demonstrate assessment procedures to study participants.
Home environments are potentially unfit for motor function assessment.	Assess home environment prior to the assessment visit. Bring standard equipment to assessment visit.	Know the home environment in advance. Ask participants about their perceived safety prior to each test.
Interviewers may encounter variations in older adults’ functional competence and home environment; the time and effort needed for each assessment visit may vary.	Identify interviewers’ skillset needed. Provide training and communicate with interviewers continuously. Pay interviewers by hour instead of by case.	Train interviewers thoroughly and continuously. Develop a learning community. Value interviewers’ contributions and incentivize appropriately.

## Data Availability

The data presented in this article are not readily available, due to privacy or ethical restrictions. De-identified participant research data presented in this article will be archived in the National Archive of Computer Data on Aging (NACDA) to advance research on aging, and will be made available to qualified researchers under a data-sharing agreement, managed by the NACDA.
